# A Three-Dimensional Spatial Metaphorical Representation of Generation Implied in Han Kin Terms

**DOI:** 10.3389/fpsyg.2021.656586

**Published:** 2021-06-03

**Authors:** Huijuan Li, Jijia Zhang, Entao Zhang

**Affiliations:** ^1^Institute of Psychology and Behavior, Henan University, Kaifeng, China; ^2^Department of Psychology, Faculty of Science and Technology, Renmin University of China, Beijing, China

**Keywords:** generation, Han kin terms, conceptual metaphor theory, three-dimensional spatial metaphor, sensorimotor system

## Abstract

Abstract concepts can be represented in the brain by means of metaphors. Generation refers to seniority in the family or clan, implies the implementation of different attitudes required by kinship, and contains profound psychological, emotional, and social factors. Generation as an abstract concept is related to concepts such as power, social status, importance, and time. The conceptual metaphor theory based on the embodied theory proposes that abstract concepts are represented by actual sensorimotor experiences. Generation implied in Han kin terms is often represented by multiple spatial terms. According to conceptual metaphor theory, the current study predicted that generation could be represented by multiple spatial metaphors. We designed six experiments to investigate this issue. The results showed that (1) the up–down and left–right positions in which kinship words were presented affected the processing of the concept of generation; (2) the processing of kinship words also affected up–down and left–right spatial information perception; and (3) the processing of the concept of generation could also automatically activate the front–back spatial operation and induce the embodied simulation of body movement. In sum, the results suggested that generation might be represented by the three-dimensional spatial metaphor of vertical, horizontal, and sagittal axes, which are influenced by the sensorimotor system.

## Introduction

An abstract concept is a concept that reflects the nature of things and the relationship between things, such as time, power, and morality. In contrast, a concrete concept refers to a concept that reflects concrete things, such as spatial orientation, length, size, and brightness. Embodied cognition theory holds that concepts are acquired through the sensorimotor activities of the body, and different sensorimotor activities leave different sensorimotor imprints in the brain (i.e., experience); human sensorimotor experience is the basis for the formation and development of concrete and abstract concepts (Barsalou, 1999, 2008, 2016; Lakoff and Johnson, 1999; Gallese and Lakoff, 2005; Pecher and Zwaan, 2005; Ye, 2010, 2011). There is extensive evidence that a concrete concept is the subject's conceptual representation of concrete things through direct sensorimotor experience (Barsalou, 2008). Abstract concepts, however, cannot be represented directly through the sensorimotor system (Ye et al., 2018); therefore, how to represent abstract concepts in the brain has been an important issue in the field of cognitive science research (Borghi et al., 2017). Conceptual metaphor theory states that abstract concepts can be represented in the brain via metaphors (Lakoff and Johnson, 1980). Metaphors map one conceptual domain (i.e., source domain) to another (i.e., target domain), the essence of which is that people use familiar and concrete representations to construct abstract and unfamiliar concepts. Research on conceptual metaphors mainly focuses on time metaphors (e.g., Boroditsky, 2000, 2018; Casasanto and Boroditsky, 2008; Fuhrman et al., 2011; Li and Zhang, 2017), power metaphors (e.g., Schubert et al., 2009; Zanolie et al., 2012; He X. et al., 2018; He and Chen, 2020), moral metaphors (e.g., Meier et al., 2007; Wang and Lu, 2013; Yin and Ye, 2014; Siev and Zuckerman, 2018), emotion (e.g., Meier and Robinson, 2004; Williams and Bargh, 2008a,b; Day and Bobocel, 2013), and so on.

### Generation Implied in Han Kinship Is a Rich Semantic Abstract Concept

Kin terms are used to represent relationships that usually refer to any blood or in-laws up to the fifth generation. According to Lévi-Straus (2006), the use of kin terms by individuals implies the implementation of different attitudes required by kinship, such as respect or closeness, rights or obligations, and affection or hostility, and contains psychological, emotional, and social factors that are more important than word appellation. In terms of kinship appellation, the Han people pay great attention to modest-respective, affinity-disaffinity, internal-external and older-younger relationships. The Han relative appellation systems are based on the “Nine generations and Wufu maps” as the basic blueprint, which include nine generations from great-great-grandfather to great-great-grandchildren and their spouses. Shi (2003) believed that the Han kin term was a narrative appellation, which was self-centered and constituted a crisscrossed system by strictly distinguishing generation, gender, blood, and degree of intimacy. Fei (1998) believed that the relationships among Chinese people were the difference pattern's network relationship based on the patriarchal clan group and centered on kinship; therefore, the Han relative appellation systems carry the rich genetic, marital, linguistic, and social and cultural information of the Han nationality.

In the basic blueprint of the Han relative appellation system, generation is one of the most important factors (Zhang and Chen, 2004; Liu et al., 2010). Generation refers to the position in the family or clan, relatives, and friends, and also refers to the rank of the lineage between the family and relatives. They are mainly divided into elders, peers, and juniors. The Han traditional culture attaches great importance to generation, which is not only reflected in folk activities, such as sacrifice, worship, the placement of spirit boards in ancestral halls, and the arrangement of genealogical names, but also in life, such as seating arrangements, greetings to elders, cigarette worship, giving up one's seat, giving way, and so forth. Generation contains profound psychological, emotional, and social attitudes, and is related to abstract concepts such as power, social rank, and importance. Generation also reflects the characteristics of age. In general, elders correspond to the previous, early, and past in terms of time, while juniors correspond to the later, late, and future; therefore, generation is an abstract concept in this perspective.

### Generation Implied in Han Kinship Terms Might Be Represented by the Three-Dimensional Spatial Metaphor

How is the generation implied in Han kinship terms represented? Jones (2010) believed that the conceptual structure of kinship seemed to borrow its organization from the conceptual structure of space. Kinship maps may utilize spatial imagery in many cultures (Leaf, 2006). Cultural kinship as a computational system is from bottom-up to top-down forms of social organization (Read, 2012). Based on the conceptual metaphor theory of embodied cognition, researchers examined the spatial metaphorical representation of generation in different ethnic groups and found that generation was metaphorically related to the concept of vertical space. For example, Li et al. (2014) explored the spatial metaphor of the Qiang kin terms for the different generations. They found that generation could be explained through the vertical space relation, and the processing of generation could also automatically guide participants' attention to the consistent position of the up–down image schema. He et al. (2015) investigated the up–down and left–right spatial metaphor of the Moso and Han kin terms. They found that (1) the kin term for the different generation and space vertical dimension existed in implicit contact in both the Han and the Moso; and (2) there was no left–right metaphor consistency effect in the processing of the Han kin term for the same generation, whereas there was part of the left–right metaphor consistency effect in the processing of the Han kin term for the same generation. Wang et al. (2017) explored the spatial and weight metaphors of seniority rules in the semantic processing of the Han and the Chinese Korean kin terms for the same generation. They found that (1) there was an up–down spatial metaphor consistency effect in the processing of the Han and the Chinese Korean kin terms for the same generation; and (2) “heavy-light” weight metaphors of kin terms existed in the Chinese Korean nationality other than the Han nationality. He et al. (2015) found that the upper and lower spatial positions affected the processing of the Han kin terms for different generations. Therefore, the current study first verified the vertical spatial metaphor of generation implied in Han kin terms and further explored whether the processing of the Han kin terms for different generations could also automatically guide participants' attention to the consistent position of the up–down image schema.

Lakoff and Johnson (1980) proposed that many abstract concepts could be understood through multiple references, each of which provided a partial perspective of an abstract concept. The same abstract concept can be mapped to multiple concrete domains (Liu et al., 2018). Many studies have shown that multiple abstract concepts can be constructed based on one specific conceptual category. For example, the up–down spatial concept can be metaphorically related to abstract concepts such as time (e.g., Boroditsky, 2000; Casasanto and Boroditsky, 2008), morality (e.g., Wang and Lu, 2013; Lu et al., 2017), and power (e.g., Zanolie et al., 2012; Wu and Wang, 2014). The same abstract concept can also be represented by multiple concrete concepts. For example, time can be represented by ego- and time-moving metaphors, and time can also be represented by spatial patterns such as up–down, left–right, front–back, and plane (e.g., Boroditsky, 2000, 2018; Casasanto and Boroditsky, 2008; Boroditsky et al., 2011; Fuhrman et al., 2011; Gu and Zhang, 2012; Laudau, 2016; Hong et al., 2017; Li and Zhang, 2017; Walker et al., 2017; He D. et al., 2018). He et al. (2015) found that Han and Moso kin terms for the different generations could be represented by the up–down metaphor, but only Moso kin terms for the same generation could be represented by part of the left–right metaphor. Wang et al. (2017) found that the Chinese Korean kin terms for the same generation could be represented by the spatial and weight metaphors, but the Han kin terms for the same generation could only be represented by the spatial metaphor. The generation that is implied in Han kin terms is not only closely related to vertical spatial orientation, but also closely related to the horizontal and sagittal spatial orientations under the influence of cultural traditions and time characteristics. In the Han traditional culture, the left is taken as the top and they consider the left as the head. The elders have high social status and high power; thus, they are usually arranged on the left in daily life and major events such as ceremonial occasions, sacrifices, and worship; juniors have lower social status and less power, and are therefore usually arranged on the right. In addition, the Han traditional culture also attaches great importance to the front and back sequence and position. For example, the elders usually have the priority right of expression and decision-making in daily life and at major events. During major events such as ceremonial occasions, sacrifices, and worship, the elders are usually placed at the front, and the juniors are usually placed at the back. The tablets of the same clan are usually arranged according to their generations, with the elder in the front and the junior in the rear. In daily language, there are many expressions to describe generations by using left–right and front–back, such as reserve the honored post for the left (虚左以待), respect for the left, right for the times (左为尊,右为次), glorify one's forefathers and enrich one's posterity (光前裕后), take over from the past and set a new course for the future (承前启后), waves on the Yangtze River before pushing waves (长江后浪推前浪), and the rising generation (后起之秀) (Lu, 2011). Furthermore, generation also reflects temporal information as self-reference. The elders correspond to a past time, while the juniors correspond to a future time. Previous studies have found that time can be represented by different spatial patterns, such as up–down, left–right, and front–back (e.g., Boroditsky, 2000, 2018; Santiago et al., 2007; Casasanto and Boroditsky, 2008; Casasanto et al., 2010; Fuhrman et al., 2011; Gu and Zhang, 2012; Ulrich et al., 2012; Hong et al., 2017; Li and Zhang, 2017; Walker et al., 2017); therefore, this study expected that generation as implied in Chinese kin terms could also be represented by the lateral (left–right) and sagittal (front–back) spatial schemas.

### Research Paradigm of Spatial Metaphor

**Figure 1:**
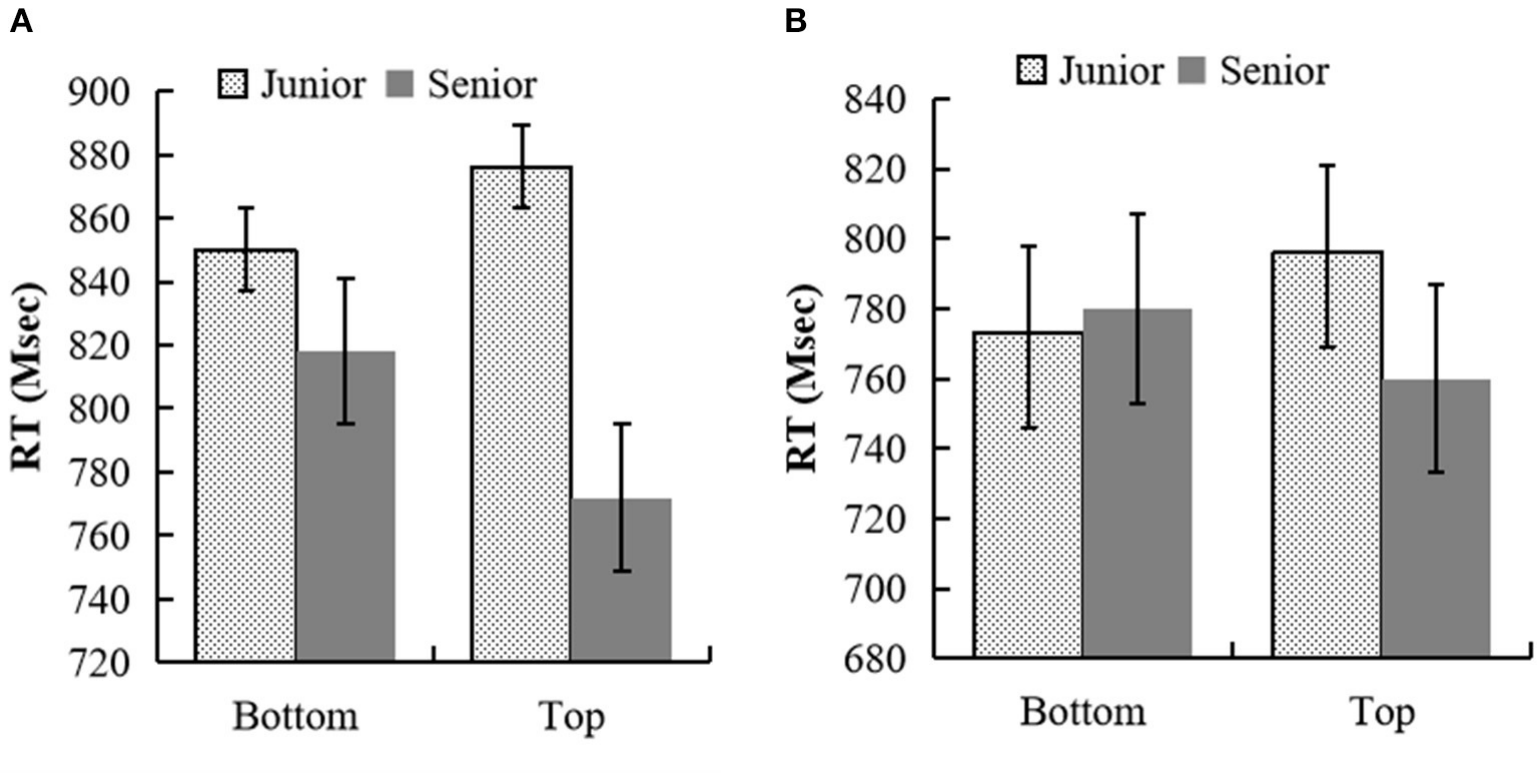
**(A)** RTs (in proportions) for the semantic judgment task of kin terms in experiment 1a. **(B)** RTs (in proportions) for the target letter identification task (p–q judgment) in experiment 1b.

Spatial Stroop paradigm is usually used to study spatial metaphor mapping from the origin domain (spatial concept) to the target domain (abstract concept). By analyzing the reaction times and error rates of words presented at different spatial locations, spatial information has consistent mental representation with specific lexical meanings in semantic judgment tasks. The spatial cluing paradigm is usually used to study the mapping from the target domain (abstract concept) to the origin domain (spatial concept). This paradigm introduces visual spatial attention as an indicator of automatic activation of the perceptive motion system. Previous studies have used similar paradigms to determine the metaphorical relationship among abstract concepts, such as morality, social status, power, generation, and time, and spatial concepts (e.g., Meier and Robinson, 2004; Zanolie et al., 2012; Ijzerman et al., 2013; Wang and Lu, 2013; Wu and Wang, 2014; He D. et al., 2018). In addition, we also referenced and combined the research procedures of Sell and Kaschak (2011) and Walker et al. (2017). Sell and Kaschak (2011) investigated the functional role of the back-front mental timeline in the processing of linguistic information. Scheifele et al. (2018) replicated Sell and Kaschak's procedure (2011) and found that the timeline is automatically activated when processing temporal sentence information, especially when the time shift is large. Walker et al. (2017) used visual presentation and manual responses to explore the different ways that space was employed when reasoning about deictic (past/future relationships) and sequential (earlier/later relationships) time. They asked participants to hold one mouse directly in front of their body and the other behind their back (see Figure 1B) for the sagittal axis.

### Overview of the Current Study

In summary, we designed six experiments to investigate the three-dimensional spatial metaphor of generation implied in Han kin terms. In experiments 1a and 1b, we used spatial Stroop paradigm and the spatial cluing paradigm explored the mapping for vertical spatial metaphors of generation implied in Han kin terms. In experiments 2a and 2b, we used similar paradigms to explore the mapping for horizontal spatial metaphors of generation implied in Han kin terms. In experiments 3a and 3b, the combined research procedures of Sell and Kaschak (2011) and Walker et al. (2017) are used to explore whether generation could be represented through the front–back spatial metaphor.

## Experiments 1A and 1B: Mapping for Vertical Spatial Metaphors of Generation implied IN Han Kin Terms

### Participants

Prior to the experiment, we conducted an *a priori* sample size calculation by using G Power (Faul et al., 2007). Assuming an alpha (α) of 0.05 and power of 0.80, the projected sample size needed to detect a medium effect size (*f* = 0.25) was determined to be 24 (ANOVA, repeated measures, within factors, 2 ×2 two-factor within-subjects design).

Accordingly, 32 Han college students participated in experiment 1a (17 females; mean age 20.42 years) and another 32 Han college students participated in experiment 1b (16 females; mean age 20.18 years) for some payment. All of them were right-handed, Mandarin Chinese speakers, and had a normal or corrected-to-normal vision and did not have a history of any psychiatric or neurological disorders. The study was approved by the local ethics committee, and all participants offered written informed consent before starting the experiment.

### Materials

Sixty-four kin terms were selected from the Chinese kin terms used by Zhang and Chen (2001) and used in daily life. Thirty-two words referred to elder kin terms (e.g., father, mother, grandpa), while another thirty-two referred to junior kinship terms (e.g., son, daughter, grandson) (see Appendix 1). The quantity of Chinese kin terms is limited; there were some kin terms with the same meaning, such as the meanings of “外公,” “姥爷,” and “外祖父” are the mother's father in elder kin terms, and the meanings of “女儿” and “闺女” are daughter in junior kin terms. The average word length (*M* = 2.44 ± 0.56) of junior kin terms was significantly higher than that of the elder kin terms (*M* = 2.13 ± 0.34), *t*_(1, 62)_ = −2.69, *P* = 0.01, Cohen's *d* = −0.67. There was no significant difference between the average stroke number of junior kin terms (*M* = 15.5 ± 5.52) and that of elder kin terms (*M* = 14.78 ± 4.03), *t*_(1, 62)_ = −0.6, *p* = 0.554, Cohen's *d* = −0.15. According to Jia (1999) stratification theory of kinship, direct relatives were valued as 1, such as father (爸爸), mother (妈妈), son (儿子), and daughter (女儿). Two-layer relatives directly related to the direct relatives were assigned a value of 2, such as grandfather (爷爷), father's younger brother (叔叔), grandson (孙子), and brother's son (侄子). Three-layer relatives associated with oneself via two-layer relatives were assigned a value of 3, such as father's older brother's wife (伯母), father's sister's husband (姑父), granddaughter's husband (孙女婿), brother's daughter's husband (侄女婿), and so on. In addition, the six foster relatives were assigned a value of 2. Limited by the selection of experimental materials, the number of kinship layers of junior kin terms (*M* = 2.41 ± 0.76) was significantly higher than that of elder kin terms (*M* = 2.03 ± 0.54), *t*_(1, 62)_ = −2.29, *p* = 0.026, Cohen's *d* = −0.57. Before the experiment, 30 Han college students who did not participate in the formal experiment were selected to evaluate the familiarity of the experimental materials using a seven-point Likert Scale (1 = very unfamiliar; 7 = very familiar). The results showed that the average familiarity of elder kin terms (*M* = 6.54 ± 0.18) and junior kin terms (*M* = 6.51 ± 0.12) had no significant difference, *t*_(1, 62)_ = 0.82, *p* = 0.418, Cohen's *d* = 0.20, which suggested that control over the word selection of generation was effective. In addition, two other elder kin terms (阿娘 = mother, 阿爹 = father) and two junior kin terms (儿郎 = son, 千金 = daughter) were used as the practice materials. Furthermore, 68 social terms were used as filler materials, which reflect the relationship between people in social life with the exception of kinship, such as doctor, counselor, teacher, and student. In order to avoid the filler material activating the spatial metaphor of social status, a small number of words in the filler material that imply social status have different proportions of high and low social status; a lot of filler words did not involve social status, such as 闺蜜 (girlfriend), 朋友 (friend), 伙伴 (partner), 邻居 (neighbor), 记者 (reporter), 作家 (author), and so on.

### Design

A 2 (generation: elder vs. junior) ×2 (position: top vs. bottom) two-factor within-subjects design was adopted. In experiment 1a, the position referred to the position of kin terms; in experiment 1b, the position referred to the position of letters. The dependent variables were the reaction times (RTs) of semantic judgment in experiment 1a, and the RTs of semantic judgment and letter recognition in experiment 1b.

### Procedure

The experiment was conducted with E-Prime 2 Professional Software (Psychology Software Tools) in a quiet room. Experimental stimuli were presented on a 14-inch computer screen. During the experiment, participants kept their eyes 50 cm away from the screen. The formal experiment consisted of two blocks. All 64 critical words were presented once in each block; hence, twice in total. Stimuli were counterbalanced across blocks. The orders of the two blocks were counterbalanced crossing participants. All stimuli were light gray on a black background.

In experiment 1a, the specific procedure was as follows: first, the “+” fixation point of 300 ms was presented in the center of the screen, following a blank screen of 200 ms. The target word was then randomly presented at the top vs. the bottom of the screen (at 75 and 25% of the screen height, respectively). Participants were required to judge whether the target word was a kin term by pressing one of two buttons (“F” or “J”) in a fast and accurate way within 3,000 ms. The response hand (left or right) was counterbalanced across participants. The system automatically recorded the judgment results; the timing unit was MS, and the error was ±1 ms (the same as below). After a blank screen of 500 ms, the next trial was initiated.

In experiment 1b, the button composition of the participant responses was to select and mark five keys, “A, D, F, J, K,” at the same horizontal position on a standard keyboard. The difference was that the J and K buttons were respectively marked as “P” and “Q.” The specific process was as follows: first, a trial started with a fixation (a “+” sign) of 300 ms followed by a centrally presented kin term. The word remained on the screen until the participant decided whether the kin word reflected an elder or junior. Participants were instructed to respond as quickly and accurately as possible by pressing one of two keys (“D” or “F”) within 3,000 ms. After a delay of 200 ms, a centered letter (“p” vs. “q”) was presented at the top vs. the bottom of the screen (at 75 and 25% of the screen height, respectively). Participants were instructed to decide whether the letter was presented by pressing the corresponding marked button (“P” vs. “Q”) on the keyboard as quickly and accurately as possible within 2,000 ms. The response mappings for the D vs. F, and P vs. Q keys were counterbalanced across participants. However, the combination of D/F vs. P/Q keys remained on the same side (hand). After a response was detected, there was a blank interval of 500 ms, after which the next trial was initiated.

All experiments started with one practice block of 16 trials, which was conducted to familiarize participants with the tasks and response mapping.

### Results and Discussion

In experiment 1a, 5.7% of trials were removed because of errors and RTs longer than 3,000 ms to the kin terms. In addition, 7.6% of trials were removed because there were more than two standard deviations (SDs) for each participant's mean RTs in each cell of the design. One subject with more than two SDs above the mean error was excluded. The analyses were conducted across participants (denoted F1), and across items (denoted F2). The remaining RTs were entered into a two (generation: elder vs. junior) × two (position: top vs. bottom) repeated measurement ANOVA (see Figure 1A). The main effect of generation was significant: *F*_1 (1, 30)_ = 36.27, *p* < 0.001, $\eta_{\text{p}}^{2}$ = 0.55, 95% CI = [45.01, 91.21]; *F*_2 (1, 62)_ = 26.20, *p* < 0.001, $\eta_{\text{p}}^{2}$ = 0.30, 95% CI = [40.01, 91.27]. The RTs of elder kin terms were faster than that of junior kin terms. The main effect of position was not significant: *F*_1 (1, 30)_ = 1.25, *p* = 0.273, $\eta_{\text{p}}^{2}$ = 0.04, 95% CI = [−7.79, 26.59]; *F*_2 (1, 62)_ = 0.12, *p* = 0.735, $\eta_{\text{p}}^{2}$ = 0.002, 95% CI = [−15.6, 22.01]. The interaction between generation and position was significant, *F*_1 (1, 30)_ = 24.41, *p* < 0.001, $\eta_{\text{p}}^{2}$ = 0.45; *F*_2 (1, 62)_ = 26.22, *p* < 0.001, $\eta_{\text{p}}^{2}$ = 0.30. The simple effect analysis showed that the junior kin terms were discriminated faster when they were presented in the bottom position than in the top position, *p*_1_ = 0.027, 95% CI = [3.29, 49.94], *p*_2_ = 0.001, 95% CI = [18.37, 71.56]. The elder kin terms were discriminated faster when they were presented in the top position than in the bottom position, *p*_1_ < 0.001, 95% CI = [23.28, 67.56], *p*_2_ < 0.001, 95% CI = [24.78, 77.97].

In experiment 1b, 13.3% of trials were removed because of errors and RTs longer than 3,000 ms to the kin terms and longer than 2,000 ms to the target letters. In addition, 4.5% of trials were removed because they had more than two standard deviations (SDs) for each participant's mean RTs in each cell of the design. The standard for removing outliers references the research of Zanolie et al. (2012). Two subjects with more than two SDs above the mean error were excluded. The analyses were only conducted across participants. The remaining RTs were entered into a two (generation: elder vs. junior) × two (position: top vs. bottom) repeated measurement ANOVA (see Figure 1B). There was no main effect of generation or position, *F*_(1, 28)_ = 3.04, *p* = 0.092, $\eta_{\text{p}}^{2}$ = 0.1, 95% CI = [−30.19, 2.43]; *F*_(1, 28)_ = 0.07, *p* = 0.791, $\eta_{\text{p}}^{2}$ = 0.003, 95% CI = [−16.87, 12.97]. Only the interaction between generation and letter present position was significant, *F*_(1, 28)_ = 8.4, *p* = 0.007, $\eta_{\text{p}}^{2}$ = 0.23. The simple effect analysis showed that the generation affected letter recognition: letters were discriminated faster in the bottom position than in the top position when they were preceded by junior kin terms, *p* = 0.013, 95% CI = [5.42, 42.1]; there was no significant difference in RTs of letter recognition in different positions when they were preceded by elder kin terms, *p* = 0.103, 95% CI = [−4.30, 44.03].

The results of experiments 1a and 1b show that the up–down image schema affected the processing of generation implied in Han kin terms; the processing of generation could automatically induce participants' attention to the position consistent with its metaphorical mapping and facilitate the recognition of the corresponding letters. The results of the two experiments suggest that there was a metaphorical relation between the concepts of generation and vertical space.

The results of experiment 1a show that participants responded faster to elder kin terms than junior kin terms. First, the relational layer of the elder kin terms is less than that of junior kin terms, which may lead to the processing advantage of the elder kin terms. The number of kinship layers is a reflection of the coefficient of relatedness. The coefficient of kinship, also known as the biological gene similarity ratio, reflects the ratio of two individuals with a common ancestor, who share the same genes. The number of kinship layers also reflects the frequency of social interactions between relatives. The fewer the layers of kinship, the more frequent the contact between relatives (Zhang and Chen, 2001), which influenced the typicality of kin terms. Zhang and Lin (2009) showed that the number of kinship layers was negatively correlated with the representation of kin terms, *r* = [−0.7, −0.52]. In order to test whether the processing advantage of elder kin terms was caused by the number of kinship layers, we took the number of kinship layers as the covariate, the generation as the independent variable, and RTs of semantic judgment as the dependent variable, and conducted the analysis of covariance (ANCOVA) across items. The result showed that RTs of elder kin terms were faster than that of junior kin terms, *F*_(1, 61)_ = 21.85, *p* < 0.001, $\eta_{\text{p}}^{2}$ = 0.26, 95% CI = [38.82, 89.37]; however, the effects of covariate were not significant, *F*_(1, 61)_ = 0.67, *p* = 0.417, $\eta_{\text{p}}^{2}$ = 0.01, 95% CI = [−11.78, 28.04]. The results suggested that the number of kinship layers had no significant effect on the semantic judgment of kin terms. In addition, the word length of elder kin terms was also less than that of junior kin terms. Thus, we took the word length of kin terms as the covariate, the generation as the independent variable, and the RTs of semantic judgment as the dependent variable, and conducted the analysis of covariance (ANCOVA) across items. The result showed that RTs of elder kin terms were faster than that of junior kin terms, *F*_(1, 61)_ = 21.28, *p* < 0.001, $\eta_{\text{p}}^{2}$ = 0.26, 95% CI = [35.58, 90.04]. The effects of covariate were not significant, *F*_(1, 61)_ = 0.41, *p* = 0.523, $\eta_{\text{p}}^{2}$ = 0.01, 95% CI = [−19.12, 37.24]. The results suggested that the word length of kin terms also had no significant effect on the semantic judgment of kin terms. Therefore, a possible reason for the processing advantage of elder kin terms is that participants are college students, unmarried, and childless. Although there was no significant difference in their judgment of familiarity with the kinship words for different generations, they had more frequent contact and interaction with elders in specific life situations and had richer sensorimotor experiences with their elders.

## Experiments 2A and 2B: Mapping for Horizontal spatial metaphors of Generation Implied in Han Kin terms

### Participants

According to the same *a priori* sample size calculation as experiment 1, 30 Han college students participated in experiment 2a (15 females; mean age 20.25 years) and another 35 Han college students participated in experiment 2b (18 females; mean age 20.12 years) for some payment. These students did not participate in other experiments. Other information related to participants was the same as for experiment 1.

### Materials, Design, and Procedure

Materials were identical to those in experiment 1. The two-factor within-subjects design was adopted: 2 (generation: elder vs. junior) ×2 (position: left vs. right). In experiment 2a, the position referred to the position of kin terms; in experiment 2b, the position referred to the position of letters. The dependent variables were the RTs of semantic judgment in experiment 2a, and the RTs of semantic judgment and letter recognition in experiment 2b.

Experiment 2 used the same instrument as experiment 1.

The procedure of experiment 2a was the same as in 1a, except that the stimulus was presented to the left (25%) or right (75%) of the horizontal center line of the computer screen.

The procedure of experiment 2b was similar to experiment 1b, with two differences: first, the J vs. K keys were marked as “W” and “M,” respectively. Second, the letters identified by participants were “M” or “W” displayed on the left (25%) or right (75%) of the horizontal center line of the computer screen. Participants were instructed to decide as quickly and accurately as possible whether a “W” or “M” was presented by pressing one of the two keys labeled “W” or “M” on the keyboard. The response mappings for the D/F and W/M keys were counterbalanced across participants; however, the combination of D/F vs. W/M keys always remained on the same side (hand).

### Results and Discussion

**Figure 2:**
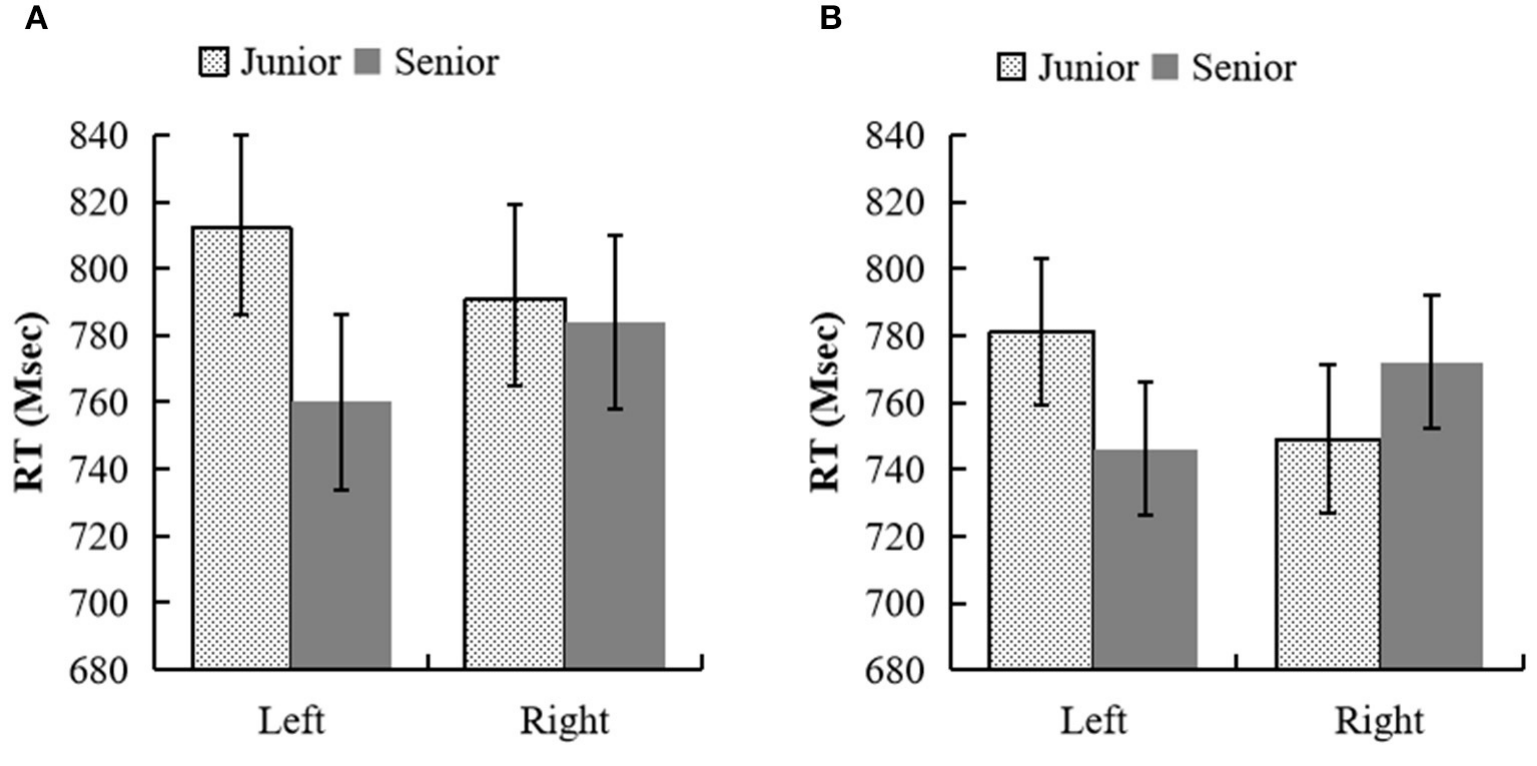
**(A)** RTs (in proportions) for the semantic judgment task of kin terms in experiment 2a. **(B)** RTs (in proportions) for the target letter identification task (m–w judgment) in experiment 2b.

In experiment 2a, 5.2% of trials were removed because of RTs longer than 3,000 ms to the kin terms. In addition, 6.6% of trials were removed because there were more than two standard deviations (SDs) for each participant's mean RTs in each cell of the design. Two subjects with more than two SDs above the mean error were also excluded. The analyses were conducted across participants (denoted F1) and across items (denoted F2). The remaining RTs were entered into a two (generation: elder vs. junior) × two (position: left vs. right) repeated measurement ANOVA (see Figure 2A). The main effect of generation was significant, *F*_1 (1, 27)_ = 7.54, *p* = 0.011, $\eta_{\text{p}}^{2}$ = 0.22, 95% CI = [7.32, 50.61]; *F*_2 (1, 62)_ = 4.12, *p* = 0.047, $\eta_{\text{p}}^{2}$ = 0.06, 95% CI = [0.33, 45.32]. RTs of elder kin terms were faster than that of junior kin terms. The main effect of position was not significant: *F*_1 (1, 27)_ = 0.15, *p* = 0.698, $\eta_{\text{p}}^{2}$ = 0.01, 95% CI = [−11.80, 8.02]; *F*_2 (1, 62)_ = 0.01, *p* = 0.911, $\eta_{\text{p}}^{2}$ < 0.001, 95% CI = [−14.92, 16.70]. The interaction between generation and position was significant, *F*_1 (1, 27)_ = 7.73, *p* = 0.010, $\eta_{\text{p}}^{2}$ = 0.22; *F*_2 (1, 62)_ = 9.37, *p* = 0.003, $\eta_{\text{p}}^{2}$ = 0.13. The simple effect analysis showed that the junior kin terms were discriminated faster when they were presented in the right side position than in the left side position, *p*_1_ = 0.038, 95% CI = [1.21, 39.58], *p*_2_ = 0.028, 95% CI = [2.74, 47.45], and the elder kin terms were discriminated faster when they were presented in the left side position than in the right side position, *p*_1_ = 0.016, 95% CI = [4.97, 43.39], *p*_2_ = 0.041, 95% CI = [0.95, 45.67].

In experiment 2b, 9.9% of trials were removed because of errors and RTs longer than 3,000 ms for the kin terms and longer than 2,000 ms for the target letters. In addition, 4.5% of trials were removed because there were more than two standard deviations (SDs) for each participant's mean RTs in each cell of the design. Two subjects with more than two SDs above the mean error were excluded. The analyses were only conducted across participants. The remaining RTs were entered into a two (generation: elder vs. junior) × two (position: left vs. right) repeated measurement ANOVA (see Figure 2B). There was no main effect of generation or position, *F*_(1, 32)_ = 0.65, *p* = 0.427, $\eta_{\text{p}}^{2}$ = 0.02, 95% CI = [−21.89, 9.49]; *F*_(1, 32)_ = 0.09, *p* = 0.766, $\eta_{\text{p}}^{2}$ = 0.003, 95% CI = [−14.8, 19.92]. Only the interaction between generation and letter present position was significant, *F*_(1, 32)_ = 11.26, *p* = 0.002, $\eta_{\text{p}}^{2}$ = 0.26. The generation affected letter recognition: letters were discriminated faster in the right side position than in the left side position when they were preceded by junior kin terms, *p* = 0.008, 95% CI = [9.1, 55.02]; letters were discriminated faster in the left side position than in the right side position when they were preceded by elder kin terms, *p* = 0.049, 95% CI = [0.16, 53.72].

The results of experiment 2 show that the left–right image schema also affected the processing of generation implied in Han kin terms; the processing of generation also activated the left and right spatial information through the metaphor mapping mechanism. The results of experiments 2a and 2b suggest that there was a metaphorical relation between the concept of generation and the concept of left–right space.

In addition, experiment 2a also found that participants responded faster to elder kin terms than junior kin terms. The results of experiment 2a indicated that the embodied sensorimotor experience to different generation relatives also affects the processing of generation.

The results of experiments 1 and 2 suggest that the concept of generation could be represented by up–down and left–right spatial schemas. Space has three-dimensional characteristics. Previous studies have found that time can be represented by different spatial patterns, such as up–down, left–right, and front–back. Could generation implied in Chinese kin terms also be represented by the front–back spatial metaphors? Experiment 3 explores whether the processing of generation implied in Han kin terms would affect the execution of the front–back position response (experiment 3a) and the execution of the forward and backward motor responses (experiment 3b).

## Experiments 3A and 3B: Mapping For Sagittal Spatial Metaphors of Generation Implied in Han Kin TERMS

### Participants

According to the same *a priori* sample size calculation as experiment 1, 30 Han college students participated in experiment 3a (15 females; mean age 20.35 years) and another 30 Han college students participated in experiment 3b (15 females; mean age 20.21 years) for some payment. These students did not participate in other experiments. Other information related to the participants was the same as in experiment 1.

### Materials, Design, and Procedure

Materials were identical to those in experiment 1. The two-factor within-subjects design was adopted: 2 (generation: elder vs. junior) ×2 (key position: front vs. back) for experiment 3a, and 2 (generation: elder vs. junior) ×2 (movement direction: forward vs. backward) for experiment 3b. The dependent variables were the RTs of semantic judgment in experiment 3a and the times between the participants' pressing down of key V to when they lifted off key V to initiate the semantic decision in experiment 3b. In addition to the dependent variable, we also recorded the times between participants' release of key V and pressing of key Z or M in experiment 3b. Consistent with previous explorations of motor compatibility effects (e.g., Glenberg and Kaschak, 2002; Sell and Kaschak, 2011; Scheifele et al., 2018), no effects were found in this measure (all F's <1). Thus, we do not discuss this variable further in this paper.

Experiment 3 used the same instrument as experiment 1a, except that the standard keyboard was oriented to the right side of the participant. The formal experiment consisted of two blocks. In one block, key Z was in front of the participant, and key M was behind the participant. In the other block, key Z was behind the participant, and key M was in front of the participant. All 64 critical words were presented once in each block (twice in total). Stimuli were random and the blocks were counterbalanced among participants. Before the formal experiment, there was a practice stage to familiarize participants with the tasks and response mapping.

The procedure of experiment 3a was as follows: first, the “+” fixation point of 300 ms was presented in the center of the screen, following a blank screen of 200 ms. The target word was then randomly presented in the center of the screen. Participants were asked to determine whether the target word was a kin term within 3,000 ms. They were asked to press key Z if the word was a kin term and key M if the word was not a kin term. Speed and accuracy were emphasized. After a blank screen of 500 ms, the next trial was initiated.

The procedure of experiment 3b was as follows: first, the “+” fixation point of 300 ms was presented in the center of the computer screen, and participants were told to hold down the V button with their right hand until the target word was randomly presented in the center of the screen. Participants were asked to determine whether the target word was a kin term. If the target word was a kin term, they were asked to release key V and move their hand to press key Z. Participants pressed all buttons with the index finger of their right hand. If the target word was not a kin term, they released key V and moved their hand to press key M. Speed and accuracy were emphasized. After a blank screen of 500 ms, the next trial was initiated.

### Results and Discussion

**Figure 3:**
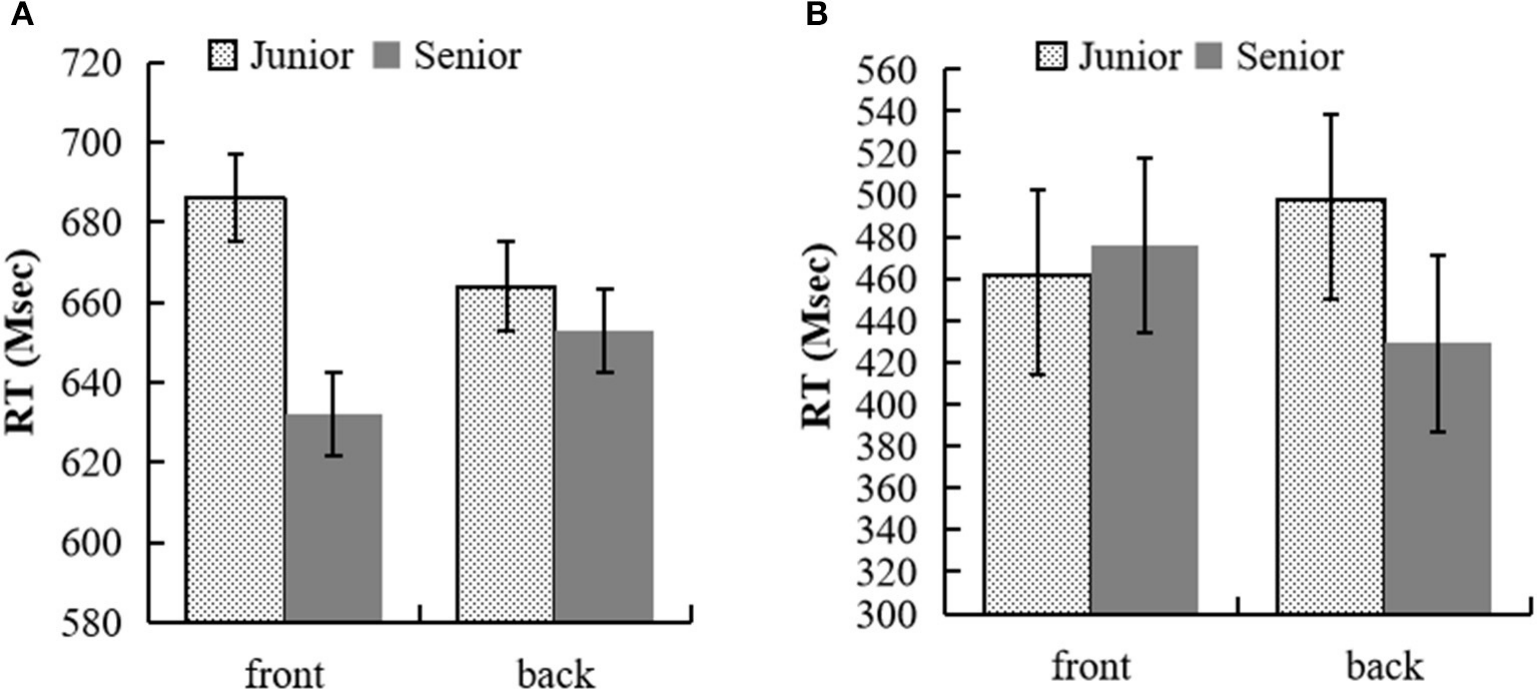
**(A)** RTs (in proportions) for the semantic judgment task of kin terms in experiment 3a. **(B)** RTs (in proportions) for initiating the sensibility judgment response in experiment 3b.

In experiment 3a, 4.2% of trials were removed because of errors and RTs longer than 3,000 ms to the kin terms. In addition, 6.4% of trials were removed because they had more than two standard deviations (SDs) for each participant's mean RTs in each cell of the design. Two subjects with more than two SDs above the mean error were excluded. The analyses were conducted across participants (denoted F1) and items (denoted F2). The remaining RTs were entered into a two (generation: elder vs. junior) × two (key position: front vs. back) repeated measurement ANOVA (see Figure 3A). The main effect of generation was significant, *F*_1 (1, 27)_ = 16.52, *p* < 0.001, $\eta_{\text{p}}^{2}$ = 0.38, 95% CI = [16.07, 48.83]; *F*_2 (1, 62)_ = 15.04, *p* < 0.001, $\eta_{\text{p}}^{2}$ = 0.2, 95% CI = [15.42, 48.21]. The RTs of elder kin terms were faster than that of junior kin terms. The main effect of position was not significant: *F*_1 (1, 27)_ = 0.01, *p* = 0.940, $\eta_{\text{p}}^{2}$ <0.001, 95% CI = [−10.76, 11.58]; *F*_2 (1, 62)_ = 0.96, *p* = 0.332, $\eta_{\text{p}}^{2}$ = 0.02, 95% CI = [−14.84, 5.09]. The interaction between generation and the button position was significant, *F*_1 (1, 27)_ = 8.30, *p* = 0.008, $\eta_{\text{p}}^{2}$ = 0.24; *F*_2 (1, 62)_ = 13.32, *p* = *0*.001, $\eta_{\text{p}}^{2}$ = 0.18. The simple effect analysis showed that participants reacted faster to junior kin terms when pressing the button behind their body than in front of their body, *p*_1_ = 0.028, 95% CI = [2.56, 40.80], *p*_2_ = 0.064, 95% CI = [−0.77, 27.40], and reacted faster to elder kin terms when pressing the button in front of their body than behind their body, *p*_1_ = 0.029, 95% CI = [2.34, 39.37], *p*_2_ = 0.002, 95% CI = [8.98, 37.15].

In experiment 3b, 3.0% of trials were removed because of errors and RTs longer than 3,000 ms for the kin terms. In addition, 6.6% of trials were removed because they had more than two standard deviations (SDs) for each participant's mean RTs in each cell of the design. One subject with more than two SDs above the mean error was excluded. The analyses were conducted across participants (denoted F1) and items (denoted F2). The remaining RTs were entered into a two (generation: elder vs. junior) × two (movement direction: forward vs. backward) repeated measurement ANOVA (see Figure 3B). The main effect of generation was significant, *F*_1 (1, 28)_ = 7.71, *p* = 0.010, $\eta_{\text{p}}^{2}$ = 0.22, 95% CI = [6.07, 40.24]; *F*_2 (1, 62)_ = 12.98, *p* = 0.001, $\eta_{\text{p}}^{2}$ = 0.17, 95% CI = [9.60, 33.54]. The main effect of position was not significant: *F*_1 (1, 28)_ = 0.44, *p* = 0.512, $\eta_{\text{p}}^{2}$ = 0.02, 95% CI = [−18.12, 35.53]; *F*_2 (1, 62)_ = 5.69, *p* = 0.02, $\eta_{\text{p}}^{2}$ = 0.08, 95% CI = [1.89, 21.53]. RTs of elder kin terms were faster than that of junior kin terms. The interaction between generation and movement direction was significant, *F*_1 (1, 28)_ = 8.53, *p* = 0.007, $\eta_{\text{p}}^{2}$ = 0.23; *F*_2 (1, 62)_ = 37.76, *p* < 0.001, $\eta_{\text{p}}^{2}$ = 0.38. The simple effect analysis showed that there was no significant difference in the movement directions for elder kin terms, *p*_1_ = 0.168, 95% CI = [−9.57, 52.34]; *p*_2_ = *0*.010, 95% CI = [4.59, 32.36]; participants reacted faster to junior kin terms when moving backward from their bodies than when moving forward from their bodies, *p*_1_ = *0*.041, 95% CI = [1.76, 75.82], *p*_1_ <0.001, 95% CI = [28.01, 55.79].

The results of experiment 3 suggest that the processing of generation implied in Han kin terms could automatically activate the execution of the front–back position response and affect the execution of the forward and backward motor responses. In addition, experiment 3 also found that the RTs of elder kin terms were faster than that of junior kin terms, indicating that the embodied sensorimotor experience for the different generation relatives also affected the execution of the front–back position response.

## Discussion

The current study investigated the psychological reality of three-dimensional spatial metaphors of generation implied in Han kin terms, namely, vertical, horizontal, and sagittal axes, and further explored the influence of three-dimensional spatial metaphors of generation on the sensorimotor system. Through six experiments, this study found that generation implied in Han kin terms could be represented by the spatial image schema of vertical, horizontal, and sagittal axes; elder kin terms had a consistent psychological representation with the spatial positions of up, left, and front, while junior kin terms had a consistent psychological representation with the spatial positions of down, right, and back. The results suggested that the three-dimensional spatial metaphorical representation of generation had psychological reality; however, He et al. (2015) found that for the same generation implied in kin terms, there was no left–right metaphor consistency effect in the semantic processing of the Han, whereas there was part of the left–right metaphor consistency effect in the semantic processing of the Moso. The difference may be due to the fact that in the feudal family of the Han nationality, the lineal son had a higher status in the family than his illegitimate elder brother. The inherited system distinguishing between lineal and concubines' children weakened the social status advantage of the elder brother to the younger brother. In addition, under the influence of the contemporary Han family size miniaturization and the only-child culture, the order of seniority among equal relatives was diluted further for Han people.

In this study, the results showed that the concept of generation could be represented by a three-dimensional spatial metaphor, and it had a stable metaphorical consistency effect. On the one hand, vertical spatial image schema could be activated automatically when the kin terms were presented in up and down spatial positions. The horizontal spatial image schema can be activated automatically when the kin terms were presented in the left and right spatial positions. The processing of the generation implied in kin terms could automatically activate the front–back spatial operation and induce the embodied simulation of body movement. At the same time, the processing of the generation could automatically activate various sensorimotor experiences. The results of this study are consistent with those of other relevant studies. The up–down spatial metaphor not only can be used to represent time, power, and morality, but also social status and emotion. Time can also be represented through different perspectives, such as self-movement or object movement, or by different spatial schemata, such as up–down, left–right, and front–back (e.g., Boroditsky, 2000, 2018; Casasanto and Boroditsky, 2008; Fuhrman et al., 2011; Gu and Zhang, 2012; Hong et al., 2017; Li and Zhang, 2017; He D. et al., 2018). The activation of multiple metaphorical representations is influenced by contextual information, and only the metaphorical representations that best fit the current situation will be activated (Torralbo et al., 2006).

In addition, the processing of generation is consistent with embodied cognition theory. According to the embodied cognition theory, abstract concepts are stored as physical experiences and extracted as embodied simulations (Barsalou, 1999, 2008, 2016; Ye, 2010, 2011, 2013, 2014; Yin et al., 2012; Zhang et al., 2013). In the specific life situation, there is a coexisting empirical relationship between the generation and the spatial position of up–down, left–right, and front–back. Since birth, people have been living in a certain space and experiencing various spatial relationships. In the process of individual growth, elder relatives tend to be larger, more capable, and caregivers, and they coexist with individual's experience of the up, left, or front. The junior relatives are usually lower in rank, weaker, and need to be cared for, and they coexist with individual's experiences of the down, right, or back. In the interaction between the body and the environment, people will establish the mapping relations between elder relatives and the sensory motor schema of upward, left, and forward, and establish the mapping relations between a junior relative and the sensory motor schema of downward, right, and backward. These mappings are stored in the channel-specific system representing abstract concepts. When the kin terms with implicit generation were presented, the corresponding sensory motor schema in the channel-specific system would be activated automatically, so as to promote the processing of the generation implied in kin terms and the recognition of target letters.

On the other hand, Han people are used to writing and reading from left to right, from top to bottom, and from front to back; thus, they tend to establish a connection between previous, early, and past time, and left, top, and front, as well as a connection between later, late, and future time and right, bottom, and back. The elders correspond in time to the past and the juniors correspond in time to the future. Previous studies have found the metaphorical consistency effect between the past time and the front, up, and left, and the metaphorical consistency effect between the future time and the back, down, and right (e.g., Boroditsky, 2000, 2018; Boroditsky et al., 2011; Sell and Kaschak, 2011; Bender and Beller, 2014; Laudau, 2016; Li and Zhang, 2017); therefore, reading and writing habits also indirectly affect the formation of the three-dimensional mapping mechanism of the generation implied in kin terms.

There may be more profound evolutionary reasons for the metaphorical connection between the generation and the front–back spatial concept. In animals, individuals have the following response to adult relatives in early life, such as the impressing effect. In daily life, parents and elders are often in front of their child, and the child follows them by looking ahead or responding forward. Children tend to be in a position behind their parents or elders, while those in front of them tend to be looking behind them. These sensorimotor experiences are stored in the mind of the individual, thus creating a connection between the elders and the front, and the juniors and the back.

In experiments 1a, 2a, and 3, the results showed that the RTs of the semantic judgment of elder kin terms were significantly faster than that of junior kin terms, which further indicated that the cognition of generation was deeply influenced by the degree of embodied sensorimotor experience. Since participants were all undergraduates, they had more frequent contact and deeper interactions with their elders than with their juniors.

Furthermore, the three-dimensional spatial metaphor of generation implied in Han kin terms is also influenced by language and culture. In the traditional Han culture, elders enjoy greater power and priority in the family. In important events such as weddings and funerals, sacrifices, and worship, people must be carried out according to their generation. In the family tree, people should be arranged from top to bottom, from left to right, and from front to back according to their generation. In Chinese, there are many words that directly describe generation by using space and time, such as ancestors vs. offspring (上辈/下辈), waves on the Yangtze River before pushing waves (长江后浪推前浪), successors (后继有人), and predecessors and descendants (前辈/后辈) (Lu, 2011). In addition, different generations have different language habits in daily communication. For example, elders tend to use words pointing to the past, such as formerly, previously, and in the past, while juniors tend to use words pointing to the future, such as afterward, later, and in future; thus, Han cultures and languages strengthen the three-dimensional spatial metaphor of generation. This view is in accordance with the hierarchical mental metaphor theory; that is, that early experience is the basis of metaphor formation, while language, culture, and other factors can strengthen specific metaphors (Casasanto and Bottini, 2014).

Unlike up–down and front–back, left–right is rarely used in language as a metaphor for generation. The left–right spatial metaphor of generation may be caused by the following: (1) it is influenced by conceptual similarity. Conceptual similarity means that the metaphorical mapping between specific concrete concepts and abstract concepts is established based on the similarity of content and structure between them (Gentner, 2003). Zhang et al. (2008) found that in the spatial word classification of Han college students, there was a cognitive theme of three-dimensional orientation around the body. In Chinese, locality words such as up, down, front, back, left, and right are generally taught to students at the same time; therefore, when participants process generation, the left–right spatial information is activated through similar concepts. (2) It is influenced by early experience and right-handedness. As mentioned earlier, children follow their elders, who also take care of them. Since humans are mostly right-handed, the elders usually use their right hands to take care of and hold children.

In summation, generation implied in Han kin terms can be represented by a three-dimensional spatial metaphor, which conforms to the strong embodiment hypothesis. The spatial metaphor of generation is influenced by physical structure, natural environment, early experience, conceptual similarity, culture, and language. Among them, body structure, natural environment, culture, and language are integrated into the specific life situation to form experience, which constitutes the basis of the three-dimensional spatial metaphor of generation. In addition, culture and experience can be expressed with the help of language to form linguistic experience, which has a strengthening effect on the three-dimensional metaphor of generation.

However, what is the relationship during three-dimensional metaphors of generation? Are the activation levels of the various metaphorical mapping mechanisms of generation consistent? What factors influence the activation of metaphor mapping of generation? Is there a metaphorical representation of generation and other spatial concepts? What is the temporal process of the metaphorical representation of the concept of generation? What about the activation of brain regions related to spatial cognition in the processing of the generation concept? All of these questions deserve further study.

## Conclusion

In general, the present study showed that the three-dimensional spatial metaphor of generation implied in Chinese kin terms has a psychological reality, and the sensorimotor system plays an important role in the processing of generation.

## Data Availability Statement

The raw data supporting the conclusions of this article will be made available by the authors, without undue reservation.

## Ethics Statement

The studies involving human participants were reviewed and approved by the Human Research Ethics Committee for Non-Clinical Faculties, School of Psychology, Henan University. The patients/participants provided their written informed consent to participate in this study.

## Author Contributions

All authors were involved in developing, editing, reviewing, and providing feedback for this manuscript and have given approval of the final version to be published.

## Conflict of Interest

The authors declare that the research was conducted in the absence of any commercial or financial relationships that could be construed as a potential conflict of interest.

## Appendix

List of Materials Used in Experiments 1, 2 and 3.

**Table:**

**Number**	**Elder**				**Junior**			
	**Chinese**	**English**	**Generation**	**层数**	**Chinese**	**English**	**Generation**	**层数**
1	爷爷	Grandfather	+2	2	儿子	Son	–1	1
2	奶奶	Grandma	+2	2	女儿	Daughter	–1	1
3	外公	Maternal grandfather	+2	2	女婿	Son-in-law	–1	2
4	外婆	Maternal grandma	+2	2	外婆	Daughter-in-law	–1	2
5	爸爸	Daddy	+1	1	孙子	Grandson	–2	2
6	妈妈	Mommy	+1	1	孙女	Granddaughter	–2	2
7	岳父	Father-in-law	+1	2	外孙	Daughter' son	–2	2
8	岳母	Mother-in-law	+1	2	外孙女	Daughter' daughter	–2	2
9	伯父	Father's older brother	+1	2	养子	Foster son	–1	2
10	伯母	Father's older brother's wife	+1	2	养女	Foster daughter	–1	2
11	舅舅	Mother's brother	+1	2	闺女	Daughter	–1	1
12	舅母	Mother's brother's wife	+1	3	姑爷	Son-in-law	–1	2
13	叔叔	Father's younger brother	+1	2	侄子	Brother's son	–1	2
14	婶婶	Father's younger brother's wife	+1	3	侄女	Brother's daughter	–1	2
15	姑父	Father's sister's husband	+1	3	孙女婿	Granddaughter's husband	–2	3
16	姑姑	Paternal aunt	+1	2	孙媳	Grandson's wife	–2	3
17	养父	Foster father	+1	2	外孙婿	Daughter' son' husband	–2	3
18	养母	Foster mother	+1	2	外孙媳	Daughter' son' wife	–2	3
19	姨父	Mother's sister's husband	+1	3	外甥	Sister's son	–1	2
20	姨妈	Maternal aunt	+1	2	外甥女	Sister's daughter	–1	2
21	姥爷	Maternal grandfather	+2	2	内侄	Son of wife's brother	–1	2
22	姥姥	Maternal grandma	+2	2	内侄女	Daughter of wife's brother	–1	2
23	外祖父	Maternal grandfather	+2	2	侄女婿	Brother's daughter's husband	–1	3
24	外祖母	Maternal grandma	+2	2	外祖母	Brother's son's wife	–1	3
25	老丈人	Father-in-law	+1	2	重孙	Great grandson	–3	3
26	丈母娘	Mother-in-law	+1	2	重孙女	Great granddaughter	–3	3
27	父亲	Father	+1	1	重外孙	Maternal great-grandson	–3	3
28	母亲	Mother	+1	1	重外孙女	Maternal great-granddaughter	–3	3
29	公公	Father-in-law	+1	2	曾孙	Great-grandson	–3	3
30	婆婆	Mother-in-law	+1	2	曾孙女	Great-granddaughter	–3	3
31	继父	Stepfather	+1	2	玄孙	Great-great-grandson	–4	4
32	继母	Stepmother	+1	2	玄孙女	Great-great-granddaughter	–4	4
**Filler item**	**Elder**				**Junior**			
1	阿爹	Father	+1	1	儿郎	Son	–1	1
2	阿娘	Mother	+1	1	千金	Daughter	–1	1
